# Supplementary material for: CRISPR-Cas9 HDR optimization: RAD52, denatured, and 5′-modified DNA templates in knock-in mice generation
Source: iScience. 2025 Oct 20;28(11):113803. doi: 10.1016/j.isci.2025.113803 (PMC12616026; doi:10.1016/j.isci.2025.113803)
Supplement: Document S1. Figures S1–S3 and Data S1–S3 [file mmc1.pdf]

iScience, Volume *28*

Supplemental information

CRISPR-Cas9 HDR optimization: RAD52, denatured, and 5′-modified DNA templates in knock-in mice generation

Boris V. Skryabin, Daniela A. Braun, Helena Kaiser, Leonid Gubar, Birte Seeger, Tasneem Khanam, Anja Stegemann, Hermann Pavenstädt, and Timofey S. Rozhdestvensky

Guide Sequence Nup_crRNA1	CTGCACTGAATTTGAGCCAG
Protospacer Adjacent Motif (PAM)	GGG
MIT Guide Specificity Score	70
Efficiency: Doench et al. 2016 Score (raw)	58
Efficiency: Doench et al. 2016 Score (percentile)	75
Efficiency: Moreno-Mateos T7 Score (raw)	84
Efficiency: Moreno-Mateos T7 Score (percentile)	98
Efficiency: Doench et al 2014 Score	16
Bae et al. Out-of-Frame Score	66

Guide Sequence Nup_crRNA2	TCAAATTCAGTGCAGAAGAT
Protospacer Adjacent Motif (PAM)	GGG
MIT Guide Specificity Score	65
Efficiency: Doench et al. 2016 Score (raw)	55
Efficiency: Doench et al. 2016 Score (percentile)	66
Efficiency: Moreno-Mateos T7 Score (raw)	36
Efficiency: Moreno-Mateos T7 Score (percentile)	31
Efficiency: Doench et al 2014 Score	29
Bae et al. Out-of-Frame Score	69

Guide Sequence Nup_crRNA8	TGTCCGTGTCTGGTCAGGGC
Protospacer Adjacent Motif (PAM)	AGG
MIT Guide Specificity Score	74
Efficiency: Doench et al. 2016 Score (raw)	52
Efficiency: Doench et al. 2016 Score (percentile)	56
Efficiency: Moreno-Mateos T7 Score (raw)	43
Efficiency: Moreno-Mateos T7 Score (percentile)	46
Efficiency: Doench et al 2014 Score	5
Bae et al. Out-of-Frame Score	67

Guide Sequence Nup_crRNA7	AAGCCTGCCCTGACCAGACA
Protospacer Adjacent Motif (PAM)	CGG
MIT Guide Specificity Score	70
Efficiency: Doench et al. 2016 Score (raw)	60
Efficiency: Doench et al. 2016 Score (percentile)	81
Efficiency: Moreno-Mateos T7 Score (raw)	54
Efficiency: Moreno-Mateos T7 Score (percentile)	69
Efficiency: Doench et al 2014 Score	29
Bae et al. Out-of-Frame Score	59

Data S1. *In silico* calculated efficiency of crRNAs used in this study.
The data were obtained from USCS BLAT mouse genome browser (mm39 assembly).

CAAGCAGGGCAGGGATATACTCATCAGTGATCAGAGCTCTTAGGATTGGCAAGGCAGA
*GG*AATTCAAGCTGGGCCCTGGTAATATAACTTCGTATAGCATACATTATACGAAGT
TATGGCTGCTGCCCAGCAGGAAACAACCTACTTGAGGGCAACCCTTCCTCATTCTG
CCTGCCAGTGCAGTCATAGCCAGGTAACTCCTCCCCTTGTACATGGTTGTCATACA
CAACCTCATTTATATGCAG**AGCATTTCTGACATGTGGGCCATGGTAAAACAAAT**
GACAGATGTGGTGCTGACACCAGCAACTGACGCCCTGAAGAGCCGGAGCAGT
GTGGAAGTGCGTATGGACTTTGTCAAGCAAGCCTTGGGATATCTTGAGCAGA
GGTAAGGAAGCTCGCTCTCTCGCTCTCTCGCTCTCTCTCTCTCTCTCTCTCTC
TCTCTCTCTCTCTCTCCAGGTTAGGATCGCATAGGATAACTTCGTATAGCATACATT
ATACGAAGTTATGGCTGCTGCCCAGCATCCTTAATGCGCGTAGTCGGATCC*GCAGG*
CTTTCACCTTCCCTAAAGTCACATGTTACTTGGTCTTACGTAGTTCTGGGACT

Data S2. DNA template sequence used in the study

The Nup93 template sequence is shown with homology arms in italics, *Lox*P sites underlined, and exon 9 highlighted in bold and underlined.

List of PCR primers used in the study:

Nup93_d1	GCATGGTAATTGGTCTGAAAGCC
Nup93_d2	CCTATAATGATGGAGGTGCTTCTGTC
Nup93_d3	GGGTGAGCCACTAAGGTTGAGC
Nup93_d4	AAGCCCTAAGAAACTTAAATAACCTTGTAA
Nup93_r1	AGAATTAGCTCAGCTCCAGTCAGTTT
Nup93_r2	GTGCAGAAACAGTCTGAGTAGATAAGTG
Nup93_r3	CACCACCCTTTCCATGTATGATTT
Nup93_r4	CGTGTTAAATTATGGAAAGCAGAGAA
LoxA1dir	AAGCTGGGCCCTGGTAAT
LoxA1rev	GCCCTCAAGTAGGTTGTTTCC
LoxA2dir	CCAGGTTAGGATCGCATAGG
LoxA2rev	CGACTACGCGCATTAAGGA

5'-end C3-spacer (C3-propyl) or 5'-end Biotin modified primers:

NUP93dB	CAAGCAGGGCAGGGATATACTCAT
NUP93rB	AGTCCCAGAACTACGTAAGACCAAGTAAC

Data S3. List of PCR primers used in this study

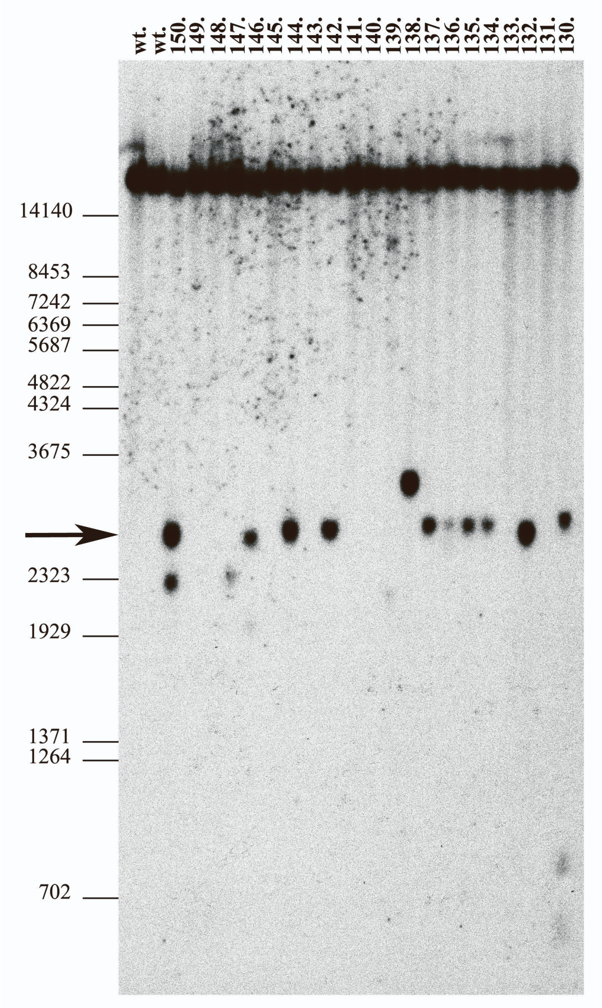

Supplementary Figure 1. Southern blot analysis of Nup93 locus targeting in FO offsprings.

DNA samples were extracted from mouse-tail biopsies of F0 offspring and digested with *BamHI*. The following groups of samples were analyzed: Samples 130–150: Microinjected with non-denatured DNA template, labelled 5'C3, and crRNA1–crRNA7 pair. Southern blot analysis was performed using a specific probe (Figure 1) following *BamHI* digestion. This detected the wild-type (*wt*) allele (21.7 kb) and two fragments (2.8 kb and 19.0 kb) corresponding to the correctly targeted allele. Notably, under the given conditions, the 21.7 kb and 19.0 kb fragments were not resolved on agarose gels (see *wt* control animals). The presence of a single 2.8 kb band in DNA samples 134 -137, 142, 144, and 146 indicates successful targeting of the *Nup93* allele. Size marker positions (in bp) are shown on the left.

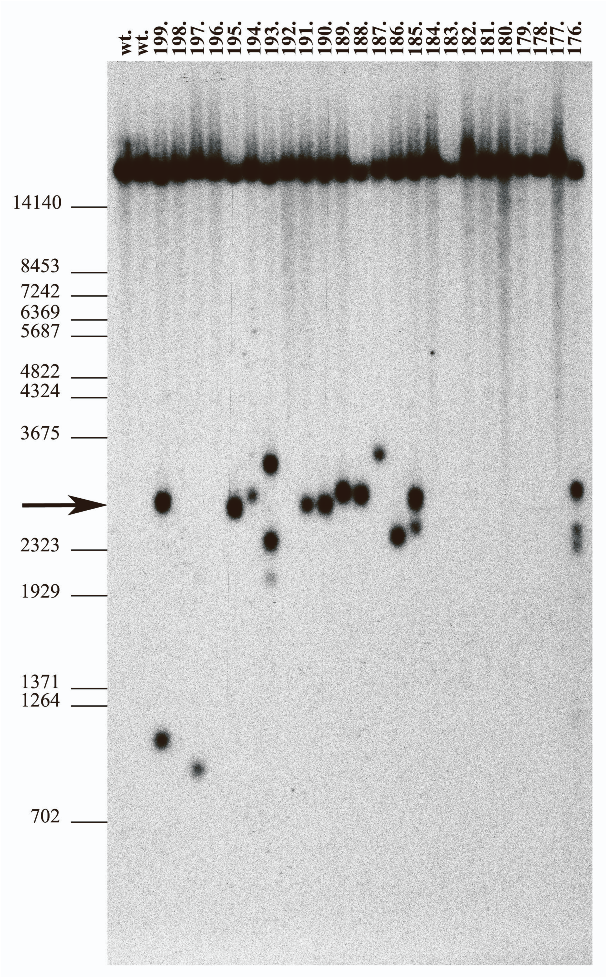

Supplementary Figure 2. Southern blot analysis of Nup93 locus targeting in FO offsprings

DNA samples were extracted from mouse-tail biopsies of F0 offspring and digested with *BamHI*. The following groups of samples were analyzed: Samples 176–183: Microinjected with denatured DNA template, labelled 5'C3, and crRNA1–crRNA7 pair. Samples 184–199: Microinjected with non-denatured DNA template, labelled 5'Biotin, and crRNA1–crRNA7 pair. Southern blot analysis was performed the same way, as described in supp. fig. 1. The presence of a single 2.8 kb band in DNA samples 188, 189, and 194 indicates successful targeting of the *Nup93* allele. Size marker positions (in bp) are shown on the left.

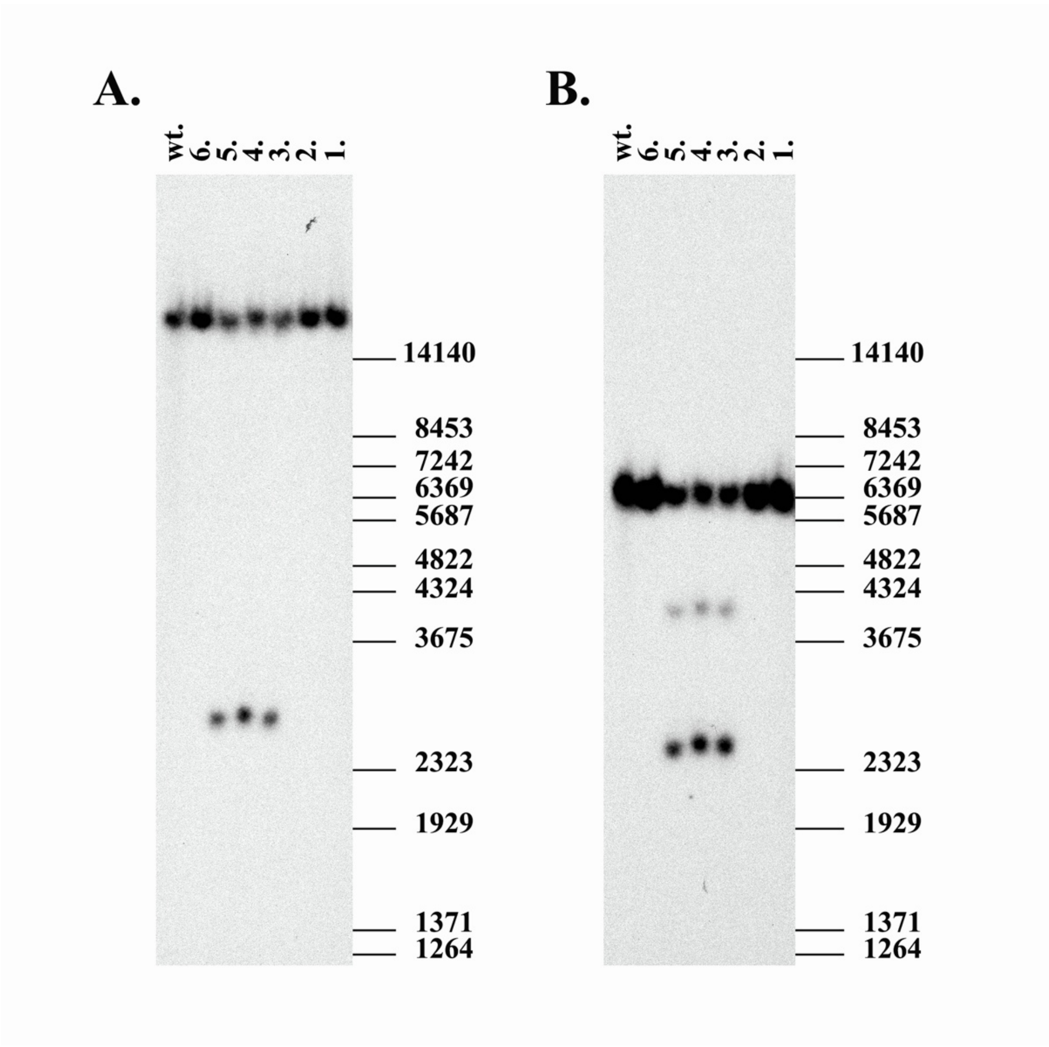

Supplementary Figure 3. **Southern blot analysis of F1 offspring**.

Southern blot analysis of genomic DNA isolated from mouse tail biopsy (numbers 1-6, and wt) and hybridized with the Nup93 HR probe. **A**: *Bam*HI enzymatic digestion revealed the wild type allele (21.7 kb) and targeted allele at (2.8 kb). DNA samples obtained from mice numbers 3 - 5 contain correctly targeted *Nup93* allele. Positions of the size marker (in bp) are shown on the right. **B**: *Eco*RI enzymatic digestion, to prove F1 mice with correctly targeted allele. The wild type allele corresponds to band 6.5 kb and targeted allele corresponds to 2.5 kb and 4.0 kb bands. DNA samples 3 - 5 contain correctly targeted *Nup93* allele.
